# The microbiota-gut-brain axis in postpartum depression: a review of microbial mechanisms and therapeutic potential

**DOI:** 10.3389/fpsyt.2026.1812091

**Published:** 2026-09-10

**Authors:** Linling Zhu, Fen Wang, Yifei Dai, Xinyun Yang, Xiudi Wang

**Affiliations:** 1 Department of Gynecology, Hangzhou Women’s Hospital, Hangzhou, Zhejiang, China; 2 Department of Gynecology, Xiaoshan District Jingjiang Subdistrict Community Health Service Center, Hangzhou, Zhejiang, China; 3 Department of Reproductive Endocrinology, Women’s Hospital, Zhejiang University School of Medicine, Hangzhou, Zhejiang, China

**Keywords:** gut-brain axis, inflammation, microbiota, postpartum depression, probiotics

## Abstract

Postpartum depression (PPD), a common but mechanistically uncertain mood disorder, occurs in 10–15% of mothers and seriously affects maternal and infant health. Recent evidence suggests that alteration of gut microbiota could dysregulate gut-brain axis functioning, which is potentially linked to the pathogenesis of PPD. This review innovatively synthesizes up-to-date preclinical and clinical evidence to illustrate relevant mechanisms, including microbial metabolite signaling, immune-inflammatory pathways, and neuroendocrine disruptors. Furthermore, the therapeutic potential of microbial-targeting approaches, including probiotics, dietary changes, and fecal microbiota transplant, to remedy PPD by restoring microbial health is discussed. Nevertheless, several notable limitations, translational uncertainties and major knowledge gaps still exist in this field. Most current evidence derives from cross-sectional observations, and well-designed longitudinal human studies are urgently needed to confirm causal relationships. In addition, available microbiota-related interventions have not yet developed into mature precision microbiome therapeutics. Against this background, this study proposes a microbiome framework connecting basic science and clinical applications that may help unravel the etiology and treatment of PPD.

## 1 Introduction

Postpartum depression (PPD) is a severe mood disorder that can have impactful consequences for the maternal-infant bond, child development, and long-term family well-being (1). Although the major impacts of PPD on society and economy—healthcare costs, loss of productivity and intergenerational effects—are well-known, we still do not know exactly how it works (2, 3). Many patients are unwilling to seek treatment due to stigma or worries about side effects. There is currently an absence of safe and effective treatment for PPD. Emerging studies suggest that dysregulation of the microbiota-gut-brain axis (MGBA) could play a pivotal role in the development of PPD, offering new insights into its biological basis (4).

According to MGBA theory, there is orderly bidirectional communication between gut microbiota and the central nervous system (CNS) (5). Immune, endocrine and neural pathways facilitate the connection. During pregnancy and after giving birth, there are some changes in hormones and the responses of the immune system, leading to substantial alterations in gut microbes (6). Specific gut dysbiosis characterized by decreased quantities of *Lactobacillus* and *Bifidobacterium* and an increased number of groups of pro-inflammatory bacteria has been observed in patients with PPD (7). The changes in microbes may lead to depressive symptoms by affecting neuroinflammation and neurotransmitters. Preclinical studies have further elucidated the role of the gut microbiota. Fecal microbiota transplantation (FMT) from depression subjects into germ-free animals induces depression-like behavior demonstrating the effect of gut microbiota on regulation of behavior (8). Microbial metabolites, especially short-chain fatty acids (SCFAs), also have come under the therapeutic spotlight for their ability to influence neuroinflammation, serotonin synthesis (9). Moreover, the translocation of endotoxins through a compromised gut barrier may lead to general inflammation towards depressive symptoms (10).

Despite these insights, significant gaps remain in our understanding of the MGBA in the context of PPD. According to this review, microbial metabolites can serve as crucial molecular mediators in the gut-brain axis (GBA) relative to PPD, and this therapeutic capability can be understood from a view of neuroimmunity. Recent advances in microbial metabolite signaling are highlighted, with an emphasis on their role in inflammation-associated synaptic dysfunction. This review also evaluates a range of emerging strategies, from precision probiotics to metabolite-targeted drugs, for their potential to reestablish harmony between the gut and the brain. This work connects mechanistic understanding with innovative treatments, seeking to transform how we approach postpartum mental health.

## 2 Overview of the MGBA

The relationship between the gut and the brain is controlled by a complex communications network between the CNS, the enteric nervous system (ENS) and the actions of the nervous, endocrine and immune systems (11). Through this axis, digestive functionality is not only controlled but it also impacts emotional regulation, cognitive processing and behavior leading to its position as a central link of metabolic activity, microbiota functionality and neuropsychiatry (12). There have been links between the gut microbiota dysbiosis and autism, anxiety-depressive behaviors, and functional gastrointestinal disorders (13). In irritable bowel syndrome, the pathogenesis is associated with a disturbance of GBA, highlighting the significance of this axis in maintaining overall health (14).

Over time, microbiome research has expanded our understanding of the GBA and its impact on mental health (15). Earliest findings set the stage for the understanding of how gut health is connected with cognitive and emotional well-being. Growing research has focused on how gut-derived microbial metabolites mediate gut-brain communication. Microbial metabolites, neuroactive compounds, and inflammatory cytokines mediate changes in blood-brain barrier permeability as well as neurogenesis and glial activity.

## 3 Alterations in the composition of gut microbiota in patients and animals with PPD

The gut microbiome’s diversity and composition undergo major changes during the postpartum period. These changes can impact the health of the mother, including the mental health. A systematic review that compiled data from the stool samples of healthy mothers showed varying alpha diversity indices but consistent compositional changes postpartum compared to the pregnancy states across studies. At the genus level, beneficial microbes like *Bifidobacterium* and *Eubacterium rectale* appeared increased after delivery, while *Akkermansia* and *Bacteroides* were rather more abundant during the pregnancy state (16). Similarly, longitudinal analyses performed in healthy women revealed distinct clustering of postpartum gut microbiota samples from pregnancy samples, with increased abundances of Actinobacteria and Firmicutes, and decreased Bacteroidetes, indicating microbial changes early in the postpartum period (17). These compositional shifts often involve a reduction in beneficial bacteria, which are critical for maintaining gut homeostasis and exerting anti-inflammatory effects. Concomitantly, there is an increased abundance of potentially pathogenic taxa, including members of Enterobacteriaceae associated with inflammatory states and dysbiosis (18, 19).

An imbalance in gut microbes is particularly concerning as research suggests that a reduction in gut microbial diversity and a shift in community profiles could worsen depressive symptoms in postpartum women. Mendelian randomization studies have identified *Bifidobacterium adolescentis* and *Coprococcus catus* as specific bacterial taxa that are inversely associated with PPD risk. However, Alphaproteobacteria and *Bilophila wadsworthia* were positively associated with depression risk, underscoring the functional relevance of these microbial fluctuations (19). Studies have shown that maternal stress and anxiety during the perinatal period may cause a more unstable and altered maternal and infant gut microbiota composition, suggesting that psychological states interact with microbial ecology (20). Influences of postpartum health on gut microbiome include maternal body mass index, gestational weight gain and dietary patterns that affect microbial diversity and taxa abundances, potentially impacting maternal metabolic and mental health outcomes (21, 22). **Table 1** demonstrates the changes in the microbiota occurring in PPD states.

**Table 1:** Microbial shifts in PPD patients and corresponding animal models.

Authors and year	Subjects	Sample size and characteristics	Postpartum time	Sequencing Platforms	Changes in microbiota	Study limitations
Zhou, Yumei et al. (23) 2020	Human	Control: n=16; PPD group: n=28	Within 1 year postpartum	16S rRNA sequencing using Illumina MiSeq	↑Actinobacteria, Bacteroidetes, Enterobacteriaceae, *Enterococcus*, *Escherichia-Shigella* ↓Firmicutes, *Faecalibacterium*, *Phascolarctobacterium*, *Butyricicoccus*, Lachnospiraceae, Lachnospiraceae, *Eubacterium-xylanophilum*, *Megasphaera*	Cross-sectional design; no causality. Small sample size. Single-center.
Liu, Zhigang et al. (24) 2020	PPD in C57BL/6J mice induced by high-fat diet	Control standard diet: n=12; HFD group: n=12	After weaning	16S rRNA sequencing using Illumina MiSeq	↑the ratio of Firmicutes and Bacteroidetes, unknown Christensenellaceae ↓unknown S24-7	Animal model, limited extrapolation. Only high-fat diet model. No causality proven.
Tian, Xin-Yun et al. (25) 2021	maternal separation stress-induced PPD model in BALB/c mice	Control: n=9; PPD group: n=10	Postpartum day	16S rRNA sequencing using Illumina HiSeq 2500 platform	↑*Eubacterium plexicaudatum*, *Eubacterium* sp. 14.2, Lachnospiraceae bacterium 3.1, *Mucispirillum schaedleri*, *Desulfovibrio piger* ↓*Bifidobacterium pseudolongum*, uncultured bacterium	Animal model. Small sample size
Zhao, Runlong et al. (26) 2022	PPD in SD rats induced by GDM	Control: n=10; Blood glucose not recovered group: n=12; Blood glucose recovered group: n=12	Early postpartum	16S rRNA sequencing using Illumina MiSeq	↑ratio of Firmicutes/Bacteroidetes, Bacteroidales, Prevotellaceae, Ruminococcaceae, Peptococcaceae 1, Coriobacteriaceae, *Prevotella*, *Lactobacillus*, *Paraprevotella* ↓Actinobacteria, Clostridiales, Lachnospiraceae, *Helicobacter*, *Clostridium XlVa*, *Ruminococcus*	Animal model. Single disease model.
Nel, Nikita H et al. (27) 2025	Human	84 stool biospecimens from female participants in the third trimester		16S rRNA sequencing using Illumina MiSeq	Higher postpartum EPDS scores tend to be associated with lower diversity, evenness, and species richness of the gut microbiome. ↑*Phascolarctobacterium*	Cross-sectional. Limited confounder control.
Xu, Shuyin et al. (28) 2025	Human	177 pregnant and 19 postpartum women	First/second/third trimester + postpartum	16S rRNA sequencing using Illumina NovaSeq 6000	↑Erysipelotrichaceae_UCG.003, *Eubacterium hallii* group, *Terrisporobacter*, *Anaerostipes*, *Blautia* ↓*Intestinimonas*, Incertae Sedis, *Eubacterium. coprostanoligenes*_group, *Coprococcus*, Christensenellaceae R.7 group	Observational, correlational; no causality. Small longitudinal subset. Chinese population only. Self-reported mood scales only.

EPDS: Edinburgh Postpartum Depression Scale; GDM: gestational diabetes mellitus; HFD: high-fat diet; PPD: postpartum depression; ↑:Increased in PPD group compared with the control group; ↓:Decreased in PPD group compared with the control group.

**Figure 1:**
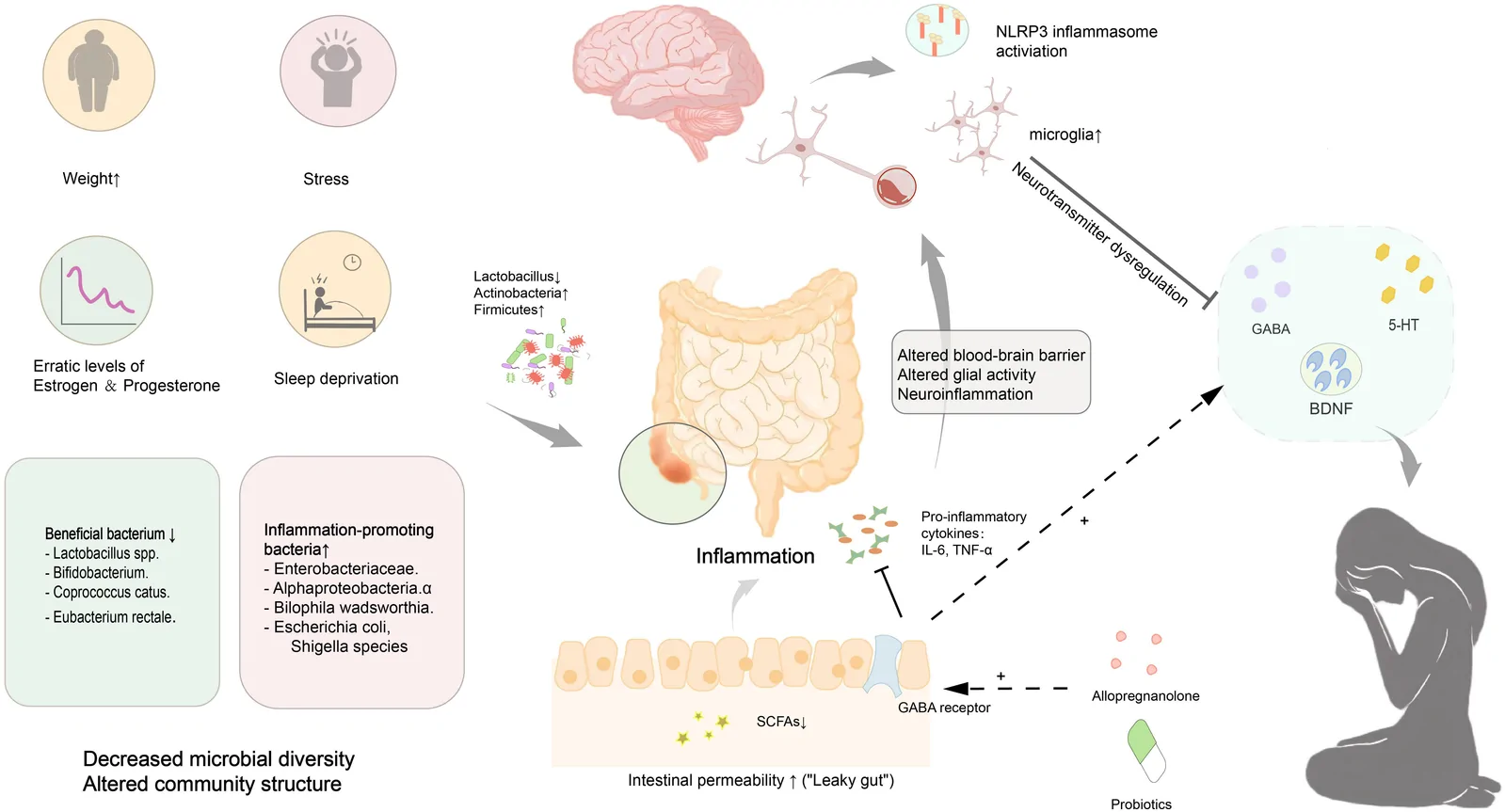
The role of gut-brain axis in PPD. Postpartum risk factors induce gut microbiota dysbiosis, leading to intestinal inflammation and elevated pro-inflammatory cytokines. These changes disrupt the blood-brain barrier, trigger neuroinflammation, and shift tryptophan metabolism away from serotonin synthesis. Concurrently, reduced microbial metabolites (GABA, SCFAs) and decreased allopregnanolone along with lower BDNF further impair CNS. Together, these interconnected pathways contribute to the development of PPD.

Notably, substantial methodological heterogeneity exists across existing human studies investigating gut microbiota in PPD, which is a key source of inconsistent microbial signatures among published reports. These variables can substantially reshape gut microbial composition and interfere with the association between gut dysbiosis and PPD risk, partially explaining inconsistent bacterial alteration results across cohorts. Detailed characteristics of included primary studies are summarized in **Table 1**. **Figure 1** depicts the role of the MGBA in the pathogenesis of PPD.

## 4 Pathogenic mechanisms of PPD associated with the MGBA

### 4.1 Neurotransmitter regulatory mechanisms in the GBA

#### 4.1.1 Regulation of the GABAergic signaling pathway

The gamma-aminobutyric acid (GABA) signaling pathway is a neurochemical system involved in the modulation of anxious and depressive behaviors, including in the context of PPD (29). The gut microbiota has a remarkable impact on GABAergic neurotransmission and emotional regulation by modulating GABA synthesis and receptor expression. Research has shown that certain gut bacteria, including *Bifidobacterium* and *Lactobacillus* species, are able to produce GABA or modify its availability in the gut, which ultimately affects the CNS by means of the GBA. The incorporation of GABA-producing probiotics has led to an elevation in GABA concentrations both in the gut and the brain, which is correlated with an alleviation of depressive and anxiety-like behaviors in animals (30–32). The GABA molecules derived from microbes may act locally on intestinal epithelial cells with GABA receptor subunit expression. They may also interfere with MAPK signaling pathways without crossing the blood-brain barrier to impact host behavior indirectly (33). Furthermore, the gut microbiota can influence the expression of GABA receptor subunits in the brain and intestine. This suggests there is a bidirectional regulatory system between peripheral microbial signals and central GABA neurotransmission (34).

In the postpartum period, changes in GABA receptor subunit expression are directly related to emotional regulation and risk of PPD. Changes of neuroactive steroids like allopregnanolone are characteristic of the postpartum period. The neurosteroids more easily target extrasynaptic GABA receptors with δ-subunits, leading to tonic inhibition and stable neuronal excitability (35, 36). The postpartum disruption of signaling via the receptor subunits can lead to defective inhibition, contributing to the pathophysiology of PPD. Clinical evidence supports this notion, as brexanolone, an intravenous formulation of allopregnanolone, has been approved by the FDA for PPD treatment, demonstrating rapid amelioration of depressive symptoms by restoring GABAA receptor function (37, 38). Zuranolone, a neuroactive steroid modulator of GABAA receptors, is also under clinical investigation for PPD, demonstrating the importance of signaling through GABA (39, 40).

The postpartum interaction between gut microbiota and GABAergic signaling fosters more than neurotransmitter production; it also strengthens receptor subunit expression and neurosteroid metabolism. Changes in the composition of gut microbes can alter systemic neuroactive steroid levels, thereby modulating GABA receptor function and emotional states (25). Experimental models such as maternal separation in rodents show increased serum GABA levels, indicating a possible role of peripheral GABA and its receptors in these behaviors (41). In addition to this, modulation of GABAergic pathways by gut microbiota might involve networks of microbial products and host systems in glutamate and serotonin, which collectively contribute to mood stabilization (42, 43).

#### 4.1.2 Serotonin metabolism and its microbial regulation

Serotonin is a neurotransmitter involved in multiple physiological functions, not only for mood regulation, and its synthesis and metabolism are strongly affected by the gut microbiota through the MGBA. Most of the serotonin in the body is produced by enterochromaffin cells in the gut, where gut microbes affect the availability and metabolism of its precursor, tryptophan. Research involving germ-free animals indicates changes in the gut serotonin metabolism; this comprises changes in tryptophan hydroxylase 1 (TPH1, the rate-limiting enzyme in peripheral serotonin synthesis) and serotonin transporter (SERT) expression (44, 45). These changes affect body systems and brain functions, which in turn affect mood and behavior.

The serotonin levels are lowered during the postpartum period. The decrease may result from gut dysbiosis that disrupts tryptophan metabolism and shifts it to the kynurenine pathway instead of serotonin synthesis. Studies on rodent models of PPD display an imbalance of their gut microbiota, reduced central and peripheral serotonin, and elevated indoleamine 2,3-dioxygenase (IDO) (26). Certain microbes, such as *Lactobacillus* and *Bifidobacterium*, may promote the production of serotonin through the increased availability of tryptophan and stimulation of TPH1, producing antidepressant effects (46, 47). Conversely, depletion of beneficial bacteria or antibiotic-induced dysbiosis lowers serotonin and worsens depressive-like behaviors (48).

Microbial metabolites, including SCFAs, modulate serotonin metabolism by influencing the activity and release of serotonin from enterochromaffin cells (49). Interventions like probiotics, prebiotics, and fecal microbiota transplantation can restore the levels of serotonin and ameliorate depression-like behavior in studies conducted on animals and clinics, underscoring the therapeutic potential of targeting microbial serotonin pathways in PPD (50).

Mechanistically, the gut bacteria modulate serotonin synthesis through controlling the expression of TPH1 and SERT, along with the availability of tryptophan and the production of bioactive metabolites. *Lactobacillus reuteri*, for instance, enhances the expression of colonic serotonin and TPH1 and inhibits other enzymes that metabolize tryptophan (46). Microbial conversion of compounds like berberine may also suppress serotonin synthesis by inhibiting TPH1, illustrating the complex role of microbes in serotonin signaling (51).

#### 4.1.3 Interaction between brain-derived neurotrophic factor and gut microbiota

BDNF is essential for neural plasticity and emotional regulation. Interestingly, the gut microbiota modulates the expression of BDNF via MGBA. Dysbiosis has been associated with decreased BDNF levels in the brain, and behavioral disturbances. Research has demonstrated that elimination of gut microbiota in animals decreases BDNF expression, contributing to mood disorder-related behavioral deficits (52, 53). Changes in the gut microbiota can result in systemic inflammation and increased intestinal permeability, which can induce neuroinflammation and disturb BDNF-mediated neuronal well-being (54).

Probiotics, including *Lactobacillus casei*, have been shown to boost BDNF expression in the brain. In animal models of depression, this probiotic increased brain levels of BDNF and activated the BDNF-ERK signaling pathway and enhanced synaptic plasticity and behavior (55). Probiotics have also been reported to rebalance gut microbiota, suppress pro-inflammatory cytokines, and improve intestinal barrier function, which are involved in the BDNF‐dependent neurogenesis and mood regulation (56).

The gut microbiota impact the BDNF at the mechanistic level through immune, metabolic and neurotransmitter pathways. Microbial molecules, such as SCFAs, enhance BDNF levels and stimulate neurogenesis (57, 58). Strategies targeting the gut microbiome, including FMT, diet, and exercise, can all enhance BDNF through modulation of gut microbiota composition, highlighting the potential of treating PPD.

### 4.2 Immunoregulation and neuroinflammation mechanisms

#### 4.2.1 Role of NLRP3 inflammasome in PPD

The neuroinflammation in PPD is mediated by NLRP3 inflammasome. In mouse models of PPD created by hormone simulation, the activation of NLRP3 in the hippocampus was associated with depressive behaviors. Reduced oxytocin receptor (OXTR) expression facilitates this activation in astrocytes, promoting neuroinflammation (59). Studies in rats have shown that there is an increase in hippocampal NLRP3, caspase-1, IL-1β, and IL-6 in PPD, while fish oil and fluoxetine reduce depressive behavior by inhibiting the NF-κB/NLRP3/caspase-1/IL-1β pathway (60). Similarly, the antidepressant effect of hypericin against PPD rats occurs via NLRP3 inhibition and regulation of glucocorticoid metabolism (61).

GBA signaling modulates the activity of NLRP3. In PPD models, withdrawal of ovarian hormones causes dysbiosis in the gut, which increases NLRP3 activation in the hippocampus to induce depressive-like behaviors. FMT from healthy donors alleviates these effects, whereas hippocampal NLRP3 overexpression abolishes the benefits of FMT, confirming NLRP3’s central role in microbiota-driven neuroinflammation (62). The prenatal stress causes depression by activating the hippocampal NLRP3 and increasing neuroinflammation in the cortex of offspring (63). Conversely, brief postpartum separation attenuates hippocampal NLRP3 and microglial activation, endowing resilience to inflammatory challenges in dams (64).

#### 4.2.2 Intestinal barrier function and systemic inflammation

The entry of lipopolysaccharide into the blood may result from increased intestinal permeability during the postpartum period. This impaired gut barrier integrity links peripheral immune activation to central inflammation and PPD. This basic inflammation interacts with neuroinflammatory pathways. Cytokines present in circulation as well as immune cells have the potential to cross or signal through the blood-brain barrier to activate microglia and thus induce neuroinflammation (65). The maintenance of the gut mucosal barrier for intestinal homeostasis is dependent on tight junction proteins (ZO-1; occludin) and antimicrobial peptides. Disruption of it eventually leads to bacterial translocation, as demonstrated by bacterial quorum-sensing molecule 3OC12 impairing barrier function and inducing systemic inflammation in models (66, 67).

In postpartum women, the intestinal integrity compromised by both endogenous hormones and stress, as evidenced by elevated serum LPS with inflammatory markers like TNF-α and IL-6 (68). This whole body inflammation negatively impacts brain function through the GBA. The use of rifaximin improves tight junction expression and reduce inflammation, suggesting therapeutic potential for PPD (69). Moreover, gut barrier dysfunction induces neutrophilia and NETs, worsening endotoxemia and barrier injury (70). The MGBA modulates barrier dysfunction and immune activations leading to neuroinflammation and depression-related behaviors (71).

#### 4.2.3 Interaction between inflammatory cytokines and neurotransmitter metabolism

The interaction between inflammatory cytokines and neurotransmitter metabolism is decisive for emotional regulation, particularly in PPD. Pro-inflammatory cytokines, such as TNF-α, IL-6, and CCL2 alter neurotransmitter systems by causing tryptophan metabolism alteration from serotonin production to kynurenine pathway via induction of indoleamine 2,3-dioxygenase (IDO) to cut down serotonin and make depressive behavior (65, 72).

The dysbiosis of gut microbiota promotes increased intestinal permeability, accumulation of LPS, and activation of TLR4 pathways, consequently exacerbating peripheral and central inflammation (72). The brain becomes inflamed and microglial cells get activated, subsequently increasing the levels of cytokines IL-1β and IL-6 which cause an impairment of serotonin, dopamine, and GABA neurotransmission (73). CCL2 also enhances recruitment of immune cells into CNS and modulates neuroinflammation, which exerts influence on neuronal function and mood (74, 75).

Treatment options targeting the interplay between cytokines and neurotransmitters, such as the use of probiotics and herbal medicines, exert antidepressant effects by enhancing the gut milieu, reducing neuroinflammation, and improving the synthesis of serotonin and dopamine (76, 77).

### 4.3 Microbial metabolites and their role in PPD

#### 4.3.1 Neuroregulatory functions of SCFAs

SCFAs, including acetate, propionate and butyrate, are the important microbial metabolite derived from dietary fiber fermentation (78–80). They impact the CNS significantly through MGBA and increase risk for PPD. Short chain fatty acids affect neuroinflammation by mediating microglial activation and cytokines production and augmenting serotonin, norepinephrine, and GABA synthesis, all of which play important roles in mood (81). In addition, butyrate is a histone deacetylase inhibitor that affects genes related to neuroplasticity and stress response (82).

Clinically, the levels of SCFA-producing bacteria, like *Lactobacillus* and *Bifidobacterium*, are reported to be reduced in women with PPD. This is coupled with reduced fecal SCFAs which correlate with increased neuroinflammation (18, 62). Maternal distress was linked with changed SCFAs levels in breast milk, which might influence infant neurodevelopment and maternal mood (83). Feces transplantation from PPD mice resulted in a reduction of SCFAs and elicited depressive-type behaviors. Restoration of the SCFA-producing bacteria has alleviated the corresponding behaviors and inhibited the activation of hippocampal NLRP3 inflammasome (62).

#### 4.3.2 Tryptophan metabolism pathways and microbial regulation

The metabolism of tryptophan is important for PPD through the MGBA. It is specifically characterized by a pathological shift from serotonin synthesis toward the pathway to kynurenine. This is evidenced by an increased kynurenine-to-tryptophan (Kyn/Trp) ratio and reduced serotonin levels in both brain regions and peripheral serum in a gestational diabetes mellitus rat model of PPD (26). Gut microbiota influence the metabolic balance by modulating the key enzymes. They up-regulate indoleamine 2,3-dioxygenase (IDO) which is the rate-limiting enzyme in the kynurenine pathway. Further, they down-regulate tryptophan hydroxylase (TPH) which is required for serotonin synthesis. These enzyme alterations are closely tied to changes in our gut microbiome, which are termed dysbiosis. One of them is reduced firmicutes to bacteroidetes ratio, along with alterations in the genera *Lactobacillus* and *Bacteroides*.

Indole derivatives along with other tryptophan metabolites are implicated in neuroinflammation as well as neurotransmission with the aryl hydrocarbon receptor among others (84). Interventions, such as probiotics that normalize the IDO/TPH expression, can restore metabolic equilibrium that lessens depression behavior (85, 86). Differences by sex exist as well. Tryptophan supplementation in mothers has different effects on the offspring. Female offspring have reduced brain tryptophan and serotonin with increased anxiety-like behaviors. The dosage increases tryptophan in the male offspring with the manifestation of repetitive behaviors. Thus, sex-dependent vulnerability of tryptophan for neurodevelopmental effect may be an important observation (87).

### 4.4 Why the MGBA may uniquely influence PPD compared with major depressive disorder

PPD emerges against a backdrop of dramatic, transient physiological changes that occur exclusively during the peripartum period. This condition is distinct from general MDD, as it interacts closely with the MGBA. Consequently, PPD creates unique pathogenic mechanisms.

Once the baby is born, balancing and stabilization of estrogen and progesterone greatly alters gut microbial composition and diversity (7). These hormones affect the functions of the gut and the levels of good bacteria including *Lactobacillus* and *Bifidobacterium*. As hormones would decline, gut homeostasis would get disrupted and dysbiosis would exacerbate along with intestinal barrier damage and the activation of peripheral inflammation and neuroinflammation, which is an initiating factor that is not very commonly seen in the main cases of MDD (88). In the postpartum period, the change of allopregnanolone disrupts GABAergic signaling. Allopregnanolone modulates extrasynaptic GABA receptors to stabilize the neuron (38). After giving birth, a decrease in this steroid affects GABA-mediated inhibition. Additionally, gut microbiota also impacts the general metabolism of allopregnanolone. The association between microbial activity and neurosteroid functionality takes place in a bidirectional manner in the PPD. Reprogramming of the immune system along with sleep deprivation and disruption of circadian rhythms are normal. After giving birth, the immune system becomes more vulnerable to the effects of bacteria. Prolonged sleep deprivation and circadian disorder exacerbate gut microbiota dysbiosis, thus creating a vicious cycle between dysbiosis, inflammation and emotional dysregulation (89, 90). The combined stressors are those which are characteristic of the postpartum state and distinguish the PPD from MDD.

Together, the overlapping effects of peripartum hormonal changes, immune remodeling, sleep disturbances, and microbial shifts result in MGBA disturbance having unique effects on the onset and progression of PPD.

## 5 Treatment strategies for PPD on the MGBA

### 5.1 Probiotic and prebiotic interventions

The use of probiotics and prebiotics shows potential as a therapeutic strategy for managing PPD by regulating gut microbiota and further modulating the MGBA. Different probiotic strains have been studied for their antidepressant effects in PPD. *Lactobacillus casei* administration in a rat model of PPD improved depressive-like behaviors, normalized gut microbiota composition, and regulated brain monoamines and oxidative stress, likely via the BDNF-ERK1/2 signaling pathway (91). Likewise, *Lactobacillus rhamnosus* HN001 has shown stress-sensitive rat anxiolytic and antidepressant activity affecting brain gene expression of GABAergic signaling and gut microbiota profile (92, 93). In a randomized controlled trial, a combination of *Limosilactobacillus reuteri* PBS072 and *Bifidobacterium breve* BB077 increased maternal mood and breastfeeding quality postpartum (94). The effects of probiotics on mood and maternal behavior are strain-specific and may involve mechanisms such as neurotransmitter modulation and immune response via the GBA.

Prebiotics are substrates that promote the growth of beneficial gut bacteria and exhibit therapeutic potential for PPD. They have therapeutic potentials. According to a study, inulin and other dietary fibers may reduce antenatal obesity-induced PPD by changing the gut microbiota composition and increasing SCFAs production and other neuro-chemical parameters and behavior (24). The SCFAs themselves are implicated in mood regulation and altered levels SCFAs with prenatal depression (95).

Based on current available evidence, the findings reflect the present clinical and preclinical observations. Clinical evidence supports the use of probiotics and prebiotics for improving PPD symptoms. A population-based cohort study linked antibiotic and gastric acid inhibitor use during pregnancy—agents that disrupt gut microbiota—with increased PPD risk, highlighting the importance of maintaining gut microbial balance (96). Furthermore, the systematic review emphasized that probiotics and prebiotics increase serotonergic neurotransmission, modulate inflammation, and enhance neurotrophic factors, playing a significant role in the neurobiology of depression (97). However, clinical trials have yielded mixed results regarding efficacy, indicating the need for larger and better-designed studies to find the best strains, doses, and durations for treatment (98). This inconsistency also suggests that the widespread clinical application of probiotics and prebiotics for PPD remains limited at the current stage.

In summary, probiotic and prebiotic intervention modifies the gut microbiota composition and functionality, acts at the level of neurochemical pathways such as BDNF and monoamines, and mollify neuroinflammation and thus mitigate symptoms of PPD. From a prospective perspective, microbiota-targeted therapies could serve as promising hypothetical therapeutic concepts and represent important directions for future experimental exploration. The results support microbiota-targeted therapies may serve as promising adjuncts or potential alternatives to conventional interventions for PPD.

### 5.2 FMT

FMT might be a promising therapeutic option for PPD as pre-clinical studies have demonstrated its ability to modulate GBA dysfunction. The FMT from a healthy mouse into a PPD mouse reduces depressive and anxiety phenotypes as well as neuroinflammation via the hippocampal NLRP3-inflammasome pathway. This was associated with restoration of gut microbiota composition and increased SCFA levels. It also reduced pro-inflammatory signaling in the gut and brain. Hence, MGBA plays an essential role in the pathogenesis and treatment of PPD (62). Similarly, FMT from mice treated with the antidepressant 919 Syrup improved depressive behaviors in PPD mice by modulating hippocampal metabolites via gut microbial regulation, which restores beneficial microbes like *Bacteroides* and *Lactobacillus* to alleviate depressive symptoms (99).

Despite some promising preclinical findings, clinical use of FMT in PPD is still in its infancy. Research on overall depression shows that FMT can positively change depressive symptoms for six months and has a good safety profile (100, 101). Preclinical evidence suggests that FMT gradually relieve depressive symptoms, suggesting that the effect of FMT on the CNS may be mediated by gut microbiota alterations via direct or indirect pathways (102). Mechanistically, FMT restores gut microbial diversity by increasing the abundance of beneficial bacteria and reducing pro-inflammatory taxa (103). The increase of fecal SCFAs after FMT is negatively related to hippocampal astrocytic activation, suggesting that microbial metabolites may be involved in the regulation of neuroinflammation (62). Current clinical trials are limited but are beginning to explore FMT’s feasibility and safety in depression and related conditions, emphasizing the need for standardized donor screening, administration routes, and dosing regimens (104, 105).

### 5.3 Dietary regulation and lifestyle interventions

Dietary and lifestyle modifications offer accessible and holistic approaches to modulating the GBA in PPD. Diets high in fiber and fermented foods contribute positively to the gut microbiota. These benefits result in an increase in the beneficial bacteria and production of SCFAs, which positively stimulate mood while reducing depressive symptoms. postpartum dietary fiber supplementation in pregnant obese mice improved gut microbiota diversity, and elevated SCFA levels (24). Human studies also correlate higher diet quality during pregnancy with reduced depressive symptoms, suggesting that balanced nutrition positively influences maternal mental health (106). Fermented foods, which have live microbes that are involved in the fermentation process, may promote the diversity and functioning of the gut microbiota, although the evidence from randomized control trials in populations suffering from PPD is lacking.

Nutrient supplementation, especially omega-3s, is of interest for mood regulation in humans. Omega-3 fatty acids assist neurotransmitter synthesis and possess anti-inflammatory effects that may influence depression. Researches in clinical work and preclinical work have demonstrated that low levels of omega-3 increase risk for depression. Similar work indicates that supplementation with fish oil or folic acid can help reduce depression (3, 107). A randomized controlled trial combining probiotics and omega-3 fatty acids showed modest improvements in depressive symptoms during pregnancy, highlighting the potential synergistic effects of dietary components on the GBA (108).

Changes in lifestyle factors, including exercise and stress management will also impact gut microbiota and brain. Regular exercise can help enhance the composition of gut bacteria, which helps improve mood (109). Stress reduction methods might help reverse hypothalamic-pituitary-adrenal (HPA) axis dysregulation associated with changes in gut microbiota and depressive symptoms (110). The relationship among stress, gut microbiota, and mood variable highlights the need for holistic lifestyle therapies for PPD.

## 6 Conclusions and future directions

The development of PPD involves interplay between biological, psychological and environmental factors, with the GBA emerging as an important mediator. The growing evidence suggests that dysbiosis of gut microbiota and its metabolites can lead to neuroimmune and endocrine disruptions that contribute to depression. Microbial metabolites are not just passive participants but active mediators of synaptic plasticity, microglial activity, and HPA axis function linking inflammation in the periphery to malfunction in the CNS.

Despite progress made recently, several key limitations remain. Current animal models for PPD cannot fully recapitulate the complex physical and psychological stress experienced by postpartum women. Distinct gut microbiota, neuroendocrine profiles and immune responses across species also create barriers to translating animal observations into clinical practice. Most published work relies on 16S rRNA sequencing, a technique that only identifies microbial taxa instead of their actual biological functions. The field also lacks universal protocols for sample handling and bioinformatic analysis, which further lowers the overall reliability of sequencing data. Postpartum research is complicated by a wide array of interfering factors, including delivery mode, breastfeeding status, dietary habits, sleep quality and psychological stress. Many clinical studies fail to adequately control these confounders, which weakens the credibility of the association between gut dysbiosis and PPD. Findings from microbiota research have proven difficult to replicate across different cohorts. Variations in participant selection criteria, ethnic background, local diets and detection platforms are largely responsible for inconsistent results, and large multicenter longitudinal studies remain rare in this field. A large number of studies have utilized correlational data between microbial taxa and depressive symptoms without demonstrating any causality. The diversity of PPD with hormonal sensitivity, immune dysregulation and psychosocial stressors necessitates stratifying patients into defined subtypes for precision interventions. Even if probiotics coupled with diet changes show promise, the results of trials are inconsistent and require standardization of strain, dosing and outcomes. In the future, research should focus on mechanistic causation and use germ-free animals populated with microbiota from patients with PPD to explore the causal relationship between particular metabolites and behavioral deficits. Studies in humans undertaken concurrently with FMT used in controlled settings may validate therapeutic potential while allaying safety fears. The combination of metagenomics, metabolomics and neuroimaging can discover microbial “signatures” that predict outcome. It is also important to investigate microbiome-epigenome interactions, since microbial metabolites may affect DNA methylation or histone acetylation in stress-related genes during postpartum hormonal shifts. It is also important to develop engineered probiotics that produce neuroprotective metabolites or degrade pro-inflammatory substances. Finally, it is critical to investigate how perinatal microbiota interventions influence maternal mental health and offspring neurodevelopment through the pathways of vertical transmission.

Clinically, implementing findings based on microbiota will face fundamental challenges. including the diagnostics standardization, delivery modality optimization of sensitive metabolites, and ethical concern navigation for FMT donor selection. Progressing from bench to bedside will require collaboration among psychiatrists, microbiologists, and bioengineers. The key to harnessing the GBA for PPD lies in a fundamental shift: we must move away from viewing microbes as passive residents toward understanding them as active builders of mental and immune resilience. This paves the way for innovative strategies in prevention, personalization, and recovery.
